# Kaposi sarcoma posthematopoietic stem cell transplant for vacuoles, E1 enzyme, X-linked, autoinflammatory, somatic syndrome

**DOI:** 10.1016/j.jdcr.2024.12.024

**Published:** 2025-01-04

**Authors:** Dennis Chen, Ryan Stubbins, Alannah Smrke, Yen Chen Kevin Ko, Pamela J. Gardner

**Affiliations:** a University of British Columbia Faculty of Dentistry General Practice Residency, Vancouver, Canada; b Leukemia/Bone Marrow Transplant Program of British Columbia, Vancouver, Canada; c BC Cancer - Vancouver Department of Medical Oncology and University of British Columbia, Vancouver, Canada; d Vancouver General Hospital Department of Pathology, Vancouver, Canada; e BC Cancer Department of Oral Oncology/Dentistry, Vancouver, Canada

**Keywords:** anatomic pathology, autoinflammatory disease, dermatology, dermatopathology, Kaposi sarcoma, oral medicine and pathology, stem cell transplant, VEXAS syndrome

## Introduction

VEXAS (vacuoles, E1 enzyme, X-linked, autoinflammatory, somatic) syndrome is a rare X-linked autoinflammatory disease that is caused by somatic mutations of the *UBA1* gene which leads to a clonal proliferation of myeloid cells.^1^, ^2^, ^3^ The estimated prevalence of VEXAS syndrome is about 1:13,591.^2^ Inflammatory skin lesions such as neutrophilic dermatosis, leukocytoclasia, panniculitis, and livedo reticularis are common.^3^, ^4^, ^5^, ^6^ The treatment of VEXAS is in evolution, but the only curative treatment is allogeneic hematopoietic stem cell transplant (allo-HSCT).^4^, ^5^, ^6^ Kaposi sarcoma (KS) is a rare transplant-related complication caused by the human herpesvirus 8.^7^, ^8^, ^9^ This case report presents a patient who developed Kaposi sarcoma following allo-HSCT as a treatment for VEXAS syndrome.

## Case report

A 61-year-old male initially contracted a COVID-19 infection and began experiencing unintentional weight loss and subcutaneous nodular lesions. His blood test demonstrated macrocytic anemia, neutropenia, and elevated C-reactive protein. A bone marrow biopsy showed hypercellularity with granulocyte predominance and clonal evolution with an inv(2) (p23q21) abnormality. Single-gene UBA1 testing showed somatic UBA1 mutation (p.Met41Thr variant), confirming the diagnosis of VEXAS syndrome. The patient was started on prednisone which resulted in symptom resolution. He remained steroid-dependent with recurrent episodes of multisystem inflammation when tapering to a daily dose below 20 mg.

The patient eventually received an allo-HSCT from an unrelated donor with a human leukocyte antigen 10 of 10 match. Pretransplant recipient viral serology was negative for human immunodeficiency virus with IgG reactivity to cytomegalovirus (CMV), herpes simplex virus type 1, varicella-zoster virus, and Epstein-Barr virus. The donor was nonreactive to CMV but reactive to Epstein-Barr virus. Reduced-intensity conditioning was done with intravenous busulfan and fludarabine. Graft-versus-host disease (GVHD) prophylaxis using anti-thymocyte globulin (ATG), tacrolimus, and methotrexate was administered. Around day +46 after allo-HSCT, the patient developed acute GVHD of the skin and upper gastrointestinal (GI) tract that was treated with corticosteroids. By day +100, there was no clinical evidence of chronic GVHD or infection. Good graft function was observed with 80% lymphoid and 97% myeloid chimerism. There was no evidence of residual autoinflammatory symptoms related to VEXAS.

**Fig 1:**
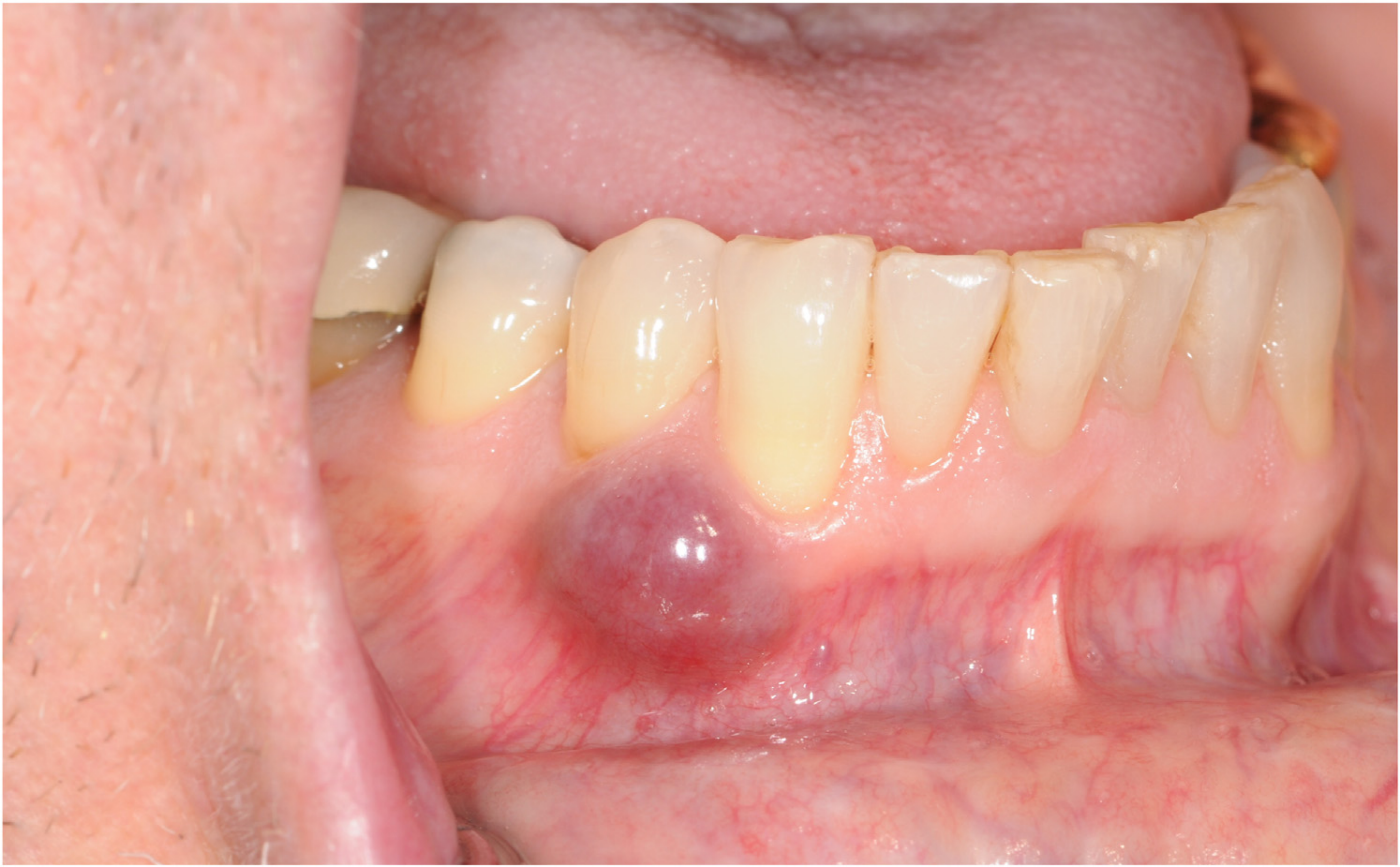
Intraoral photograph of the lesion near teeth 43 (27) and 44 (28).

**Fig 2:**
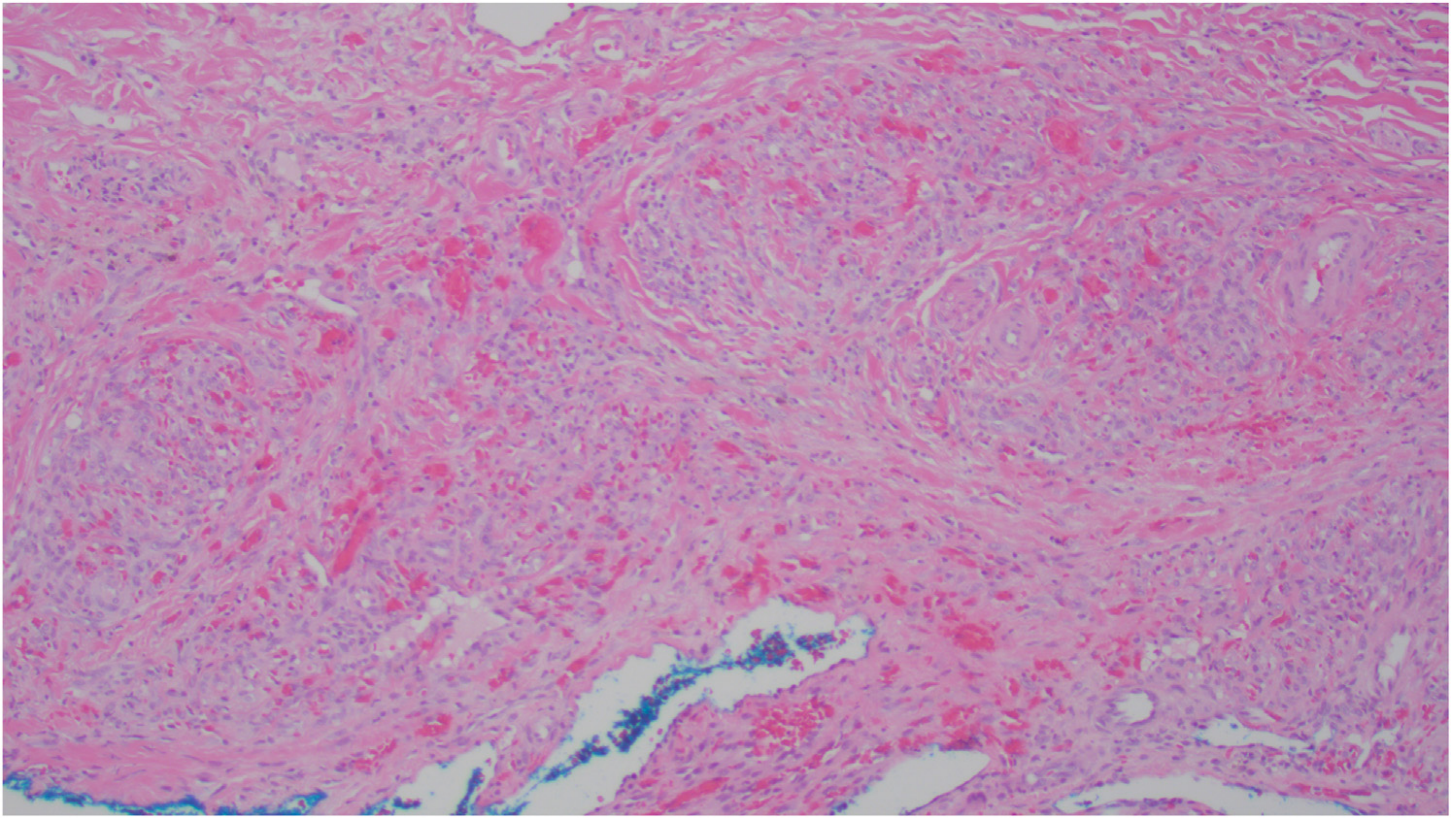
H&E stained histology slide under high-power.

**Fig 3:**
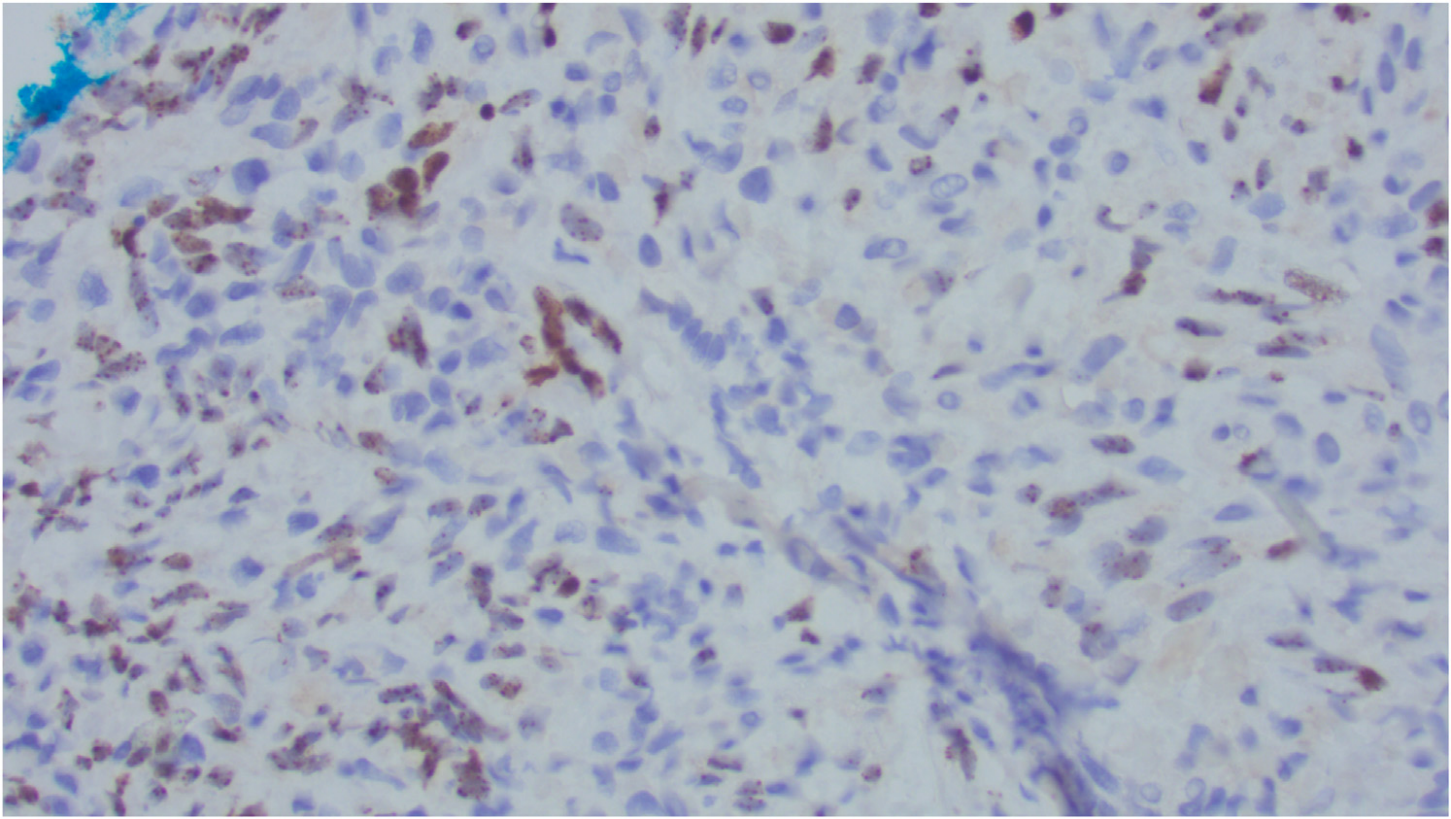
HHV-8 immunochemistry slide. HHV-8, human herpesvirus 8.

At +175 days post-transplant, the patient developed an approximate 1x1x0.5 cm raised, nonblanching, firm, nonmobile, and violaceous asymptomatic lesion on the buccal gingiva adjacent to teeth 43 (27) and 44 (28) (Fig 1). A periapical radiograph of the area showed no radiographic changes to the mandibular bone or tooth roots. Based on the clinical presentation, the differential diagnosis included myeloid sarcoma, giant cell granuloma, or pyogenic granuloma. An incisional biopsy was done. Histopathology showed squamous mucosa with subepithelial proliferation of irregularly shaped vascular spaces and extravasated erythrocytes (Fig 2). Immunohistochemistry testing was positive for human herpesvirus 8 (HHV-8) (Fig 3). The final diagnosis was KS. HIV testing was negative. Subsequent computed tomography scan of the neck to pelvis did not demonstrate any visceral involvement. Esophagogastroduodenoscopy and flexible sigmoidoscopy demonstrated a sub-centimeter KS lesion in the proximal esophagus. The management plan included: tapering of immunosuppression (cyclosporine) to reconstitute T-cell function. Upon stopping cyclosporine, the KS lesions doubled in size and the GVHD flared necessitating re-introduction of immunosuppression. Liposomal doxorubicin was thus started due to lack of regression with cyclosporine withdrawal and need for immunosuppression. With 1 cycle of liposomal doxorubicin the cutaneous KS lesions did regress, then stabilized. After cycle 1, liposomal doxorubicin was held due to severe thrombocytopenia from treatment of re-activation of CMV viremia. At time of publication, the KS lesions remain stable with no further liposomal doxorubicin being administered due to ongoing treatment of CMV.

## Discussion

This case report documents the rare occurrence of KS following allo-HSCT for VEXAS syndrome, a newly identified and rare autoinflammatory disease. Post-HSCT KS is considered iatrogenic with an estimated incidence rate of 0.05% and 0.17% for autologous and allogeneic HSCT, respectively.^7^ A 2019 retrospective cohort study found only 13 cases of post-HSCT KS were recorded in the registry of the European Society for Blood and Marrow Transplantation database from 1987 to 2017.^7^ The median time from HSCT to KS diagnosis was approximately 7 months.^8^^,^^9^ In this case report, our patient was diagnosed with KS 5 months after allo-HSCT. Similarly, VEXAS is an extremely rare autoinflammatory disease with no estimated global incidence rate.

HHV-8 causes KS by establishing a lifelong infection within the host endothelial and mesenchymal cells.^8^^,^^9^ The cardinal histopathologic presentation of KS includes immune cell infiltration, extensive angiogenesis, slit-like vascular spaces, and the proliferation of spindle cells of endothelial origin.^8^^,^^9^ Similarly, the histopathology of oral KS in this case report showed squamous mucosa with irregular vascular spaces and extravasated erythrocytes. Iatrogenic KS is associated with immunosuppression from organ or hematopoietic stem cell transplant, chemotherapy, or immunosuppressive therapies.^7^, ^8^, ^9^ Transplant-associated KS often responds to immunosuppression withdrawal.^8^^,^^9^ Switching of immunosuppressant class from calcineurin inhibitors to mammalian target of rapamycin inhibitors has also shown evidence of KS regression.^8^^,^^9^ Systemic chemotherapy is required when immunosuppression withdrawal results in no evidence of KS regression or clinically significant reactivation of conditions requiring immunosuppression.^8^^,^^9^ Cytotoxic agents such as liposomal-doxorubicin and paclitaxel may be used as treatments.^8^^,^^9^ Localized treatment such as radiation, cryotherapy, and surgical excision can also be considered.^8^^,^^9^

## Conclusion

This case report documents the occurrence of KS following allo-HSCT treatment of VEXAS syndrome. To date, there has been no documented oral manifestation of VEXAS syndrome. As both conditions are extremely rare, additional research is needed to examine oral manifestations of VEXAS syndrome and the potential pathophysiological link between VEXAS syndrome and KS.

## Conflicts of interest

None disclosed.
